# Novel Formulations Containing Fluorescent Sensors to Improve the Resolution of 3D Prints

**DOI:** 10.3390/ijms231810470

**Published:** 2022-09-09

**Authors:** Monika Topa-Skwarczyńska, Andrzej Świeży, Dominika Krok, Katarzyna Starzak, Paweł Niezgoda, Bartosz Oksiuta, Weronika Wałczyk, Joanna Ortyl

**Affiliations:** 1 Department of Biotechnology and Physical Chemistry, Faculty of Chemical Engineering and Technology, Cracow University of Technology, Warszawska 24, 30-155 Kraków, Poland; 2 Photo4Chem Ltd., Lea 114, 30-133 Kraków, Poland; 3 Photo HiTech Ltd., Bobrzyńskiego 14, 30-348 Kraków, Poland

**Keywords:** photopolymerisation, rheology, real-time FT-IR, advanced materials, 3D printing

## Abstract

Three-dimensional printing in SLA (stereolithography) and DLP (digital light processing) technologies has recently been experiencing a period of extremely rapid development. This is due to the fact that researchers recognise the many advantages of 3D printing, such as the high resolution and speed of the modelling and printing processes. However, there is still a search for new resin formulations dedicated to specific 3D printers allowing for high-resolution prints. Therefore, in the following paper, the effects of dyes such as BODIPY, europium complex, and Coumarin 1 added to light-cured compositions polymerised according to the radical mechanism on the photopolymerisation process speed, polymerisation shrinkage, and the final properties of the printouts were investigated. The kinetics of the photopolymerisation of light-cured materials using real-time FT-IR methods, as well as printouts that tangibly demonstrate the potential application of 3D printing technology in Industry 4.0, were examined. These studies showed that the addition of dyes has an effect on obtaining fluorescent prints with good resolution.

## 1. Introduction

The development of Industry 4.0 [1] which is one of the most rapidly growing branches of the economy combining digital–physical systems significantly affecting the efficiency and course of technological processes [2], as well as the growing demand for ever newer solutions, has made additive prototyping technologies more and more desirable and resulted in key methods for developing mock-up models with the characteristics and properties of the target product, i.e., physical properties, ergonomics of operation, or appearance [3,4]. Fast yet efficient and widely available methods of prototyping [5] such objects are 3D printing technologies using photopolymerisation processes [6,7,8,9,10,11,12,13,14,15,16].

Commonly used techniques in photo-curable 3D printing are SLA (stereolithography) [16], DLP (digital light processing) [17], and CLIP (continuous liquid interface production) techniques [18,19].

SLA is the earliest rapid prototyping technology with a stable printing process and numerous machine suppliers. The SLA technology uses a laser. A construction plate is moved in small increments, and a UV laser draws the cross-section layer by layer. The process repeats until a model is created. The printing resolution of this technique depends on the size of the laser beam, so, compared to other photo-curing techniques, SLA has low resolution. Moreover, SLA has low printing efficiency due to the curing speed depending on the movement of the laser beam. The larger the size of the models, the slower the printing speed. Nevertheless, the precision of the SLA technique is good enough to print objects with complex structures and small sizes. The technique is used in dentistry, toy manufacturing, automotive, and aerospace [18,20]. On the other hand, DLP technique uses a projector, such as those used for office presentations or home theatres, to project an image of a cross-section of an object onto a photosensitive liquid resin. DLP 3D printing has the advantage of printing the objects with a small size and high precision [18,21].

Another dynamically developing 3D printing technique is the so-called CLIP (continuous liquid interface production) technique. The process works in a similar scheme to DLP. In this technique, a projector is also used as a light source. In addition, the underside of the resin tub is constructed from a material permeable not only to UV radiation, but also to oxygen. Oxygen interfering with the polymerisation process just above the bottom of the tub creates a so-called dead zone. The polymerisation process in the dead zone does not occur because the oxygen concentration is too high. This effect allows the layer of liquid resin to be reconstituted as the printed object is lifted on the working platform [7,22]. This method can achieve accuracy within tenths of microns [7,18]. Undoubtedly, the CLIP technique’s most significant advantage is its printing speed. Using the dead zone in the CLIP technique makes it possible to carry out printing continuously, significantly reducing the processing time compared to the DLP technique. Nevertheless, there are still some limitations to this technique. Unfortunately, the use of oxygen inhibition limits the formulation of the applied compositions. So far, to achieve fast printing through the CLIP technique, a low viscosity resin and a hollow model are required. The former ensures that the resin is quickly delivered to the printing area, while the hollow model reduces the amount of resin required. Therefore, for solid models and high viscosity resins, the performance of the CLIP technique is low. In addition, the membrane responsible for oxygen permeation is expensive. Furthermore, the resolution achieved by the CLIP technique is of high quality, comparable to SLA technology. Consequently, the possibility of realising a high-speed printing process using the high-resolution photopolymerisation process offers new development opportunities [22].

These technologies use photochemical processes in which successive layers of appropriately prepared resin formulation are cured [23] under the influence of radiation from the wavelength range of usually 405 nm [24,25,26].

Most often, resins containing monomers polymerised according to the radical mechanism are used for photocurable 3D printing in all technologies, which provide high degrees of crosslinking and prints with very good resolution [27,28]. Nevertheless, as is known from studies by various research teams, the key aspect limiting the possibilities of printing based on crosslinking monomers according to the free radical mechanism is the volumetric polymerisation shrinkage resulting from the change in interatomic interactions from van der Waals forces to covalent bonds [29]. Therefore, new resins with reduced polymerisation shrinkage are still being sought through the introduction of inert fillers or additives such as talc, surface-modified sepiolite nanofibers, graphene oxide, and TiO_2_ and SiO_2_ nanoparticles [30,31].

Some interesting developments are the introduction of dyes into 3D printing, which give colour to 3D objects. Dyes such as coumarins [32], quinolines [33], pyridines [34] and aminophthalimide [35] have been added to resin formulations undergoing photopolymerisation processes. Initially, they have mainly been used to monitor photopolymerisation processes [36,37]. Most generally, fluorophores significantly affect parameters such as viscosity or polarity, which are changed during the photopolymerisation process [38]. Then, sensors exhibit bathochromic or hypochromic shifts, and the intensity itself is altered [39]. In studies using methods such as FPT (fluorescence probe technology), it allows accurate monitoring of the process taking place through significant changes in the nature of the fluorescence intensity spectrum of a given dye.

Nevertheless, dyes are still usually undervalued in 3D printing, because they are introduced only for aesthetic reasons [28]. It is worth noting that recently the accelerating properties of coumarins [40] or the BODIPY [41] fluorophore have also been proven, where the introduction of these fluorophores increases the speed of the photopolymerisation process. On the other hand, europium complexes have been investigated as, for example, thickness sensors during radical photopolymerisation [42], as well as a luminescent additive to a composition based on a polylactic acid polymer filled with reduced graphene oxide nanoplatelets for 3D printing [43].

Therefore, in the present paper, the effect of the dyes BODIPY, europium complex, and Coumarin 1 added to light-curing compositions polymerised according to the radical mechanism on the rate of photopolymerisation process, polymerisation shrinkage, and final properties of prints was investigated. The kinetics of photopolymerisation of light-curing materials was examined by real-time FT-IR. Polymerisation shrinkage was also measured using an Anton Paar apparatus. Moreover, printing of the developed resins was performed on printers that work with different technologies: a LumenX (from Cellink3D), working with DLP technology, Photon Mono X (from Anycubic), working with SLA technology, and a laser engraver.

## 2. Results

### 2.1. Fluorescent Dyes as a Component of Bimolecular Photoinitiating Systems for Photopolymerisation Processes

In this article, three fluorescent dyes, [4-phenyl-2,6-bis(2-pyridyl)pyridine]tris [4,4,4-trifluoro-1-(4-nitrophenyl)butane-1,3-dione]europium(III), 7-diethylamino-4-methylcoumarin (Coumarin 1), and B-1 4,4-difluoro1,3,5,7,8-pentamethyl-4-bora-3a,4a-diaza-s-indecene (BODIPY), are presented as additives to photo-curable compositions dedicated to 3D printing.

Due to the fact that previous spectroscopic studies have shown that these dyes absorb in the visible range and exhibit fluorescent properties [41,44,45], they can be excellent additives for 3D printing. The addition of dyes to formulations dedicated to 3D printing can contribute not only to improving the visual effects of the obtained objects, but also to increasing the resolution of the final prints [46].

Therefore, in order to determine the influence of the presented dyes on the final properties of printouts, basic kinetic studies of the photopolymerisation process were carried out first. These dyes were tested in binary photoinitiating systems which also contained the TPO photoinitiator commonly used in industry. Monitoring of the radical photopolymerisation of acrylate monomers (mixtures of monomer trimethylolpropane triacrylate (TMPTA), bisphenol A ethoxylate diacrylate (BEDA), and isobornyl acrylate (IBOA) in a weight ratio of 1:2:7) at a wavelength in the VIS range with an emission maximum of 405 nm was performed. The 1 wt% diphenyl(2,4,6-trimethylbenzoyl)phosphine oxide (TPO) initiator was introduced into the formulation as a photoinitiator. This initiator photodissociates were exposed to light in the visible range and thus effectively initiated the photopolymerisation process. Suitable fluorescent dyes were added to the formulation at a concentration of 0.1 wt%. The one-component photoinitiating system consisting of TPO (1 wt%) and a mixture of monomers TMPTA, IBOA, and BEDA in weight ratio of 1:2:7 without the addition of fluorescent dyes was used as a reference.

**Figure 1:**
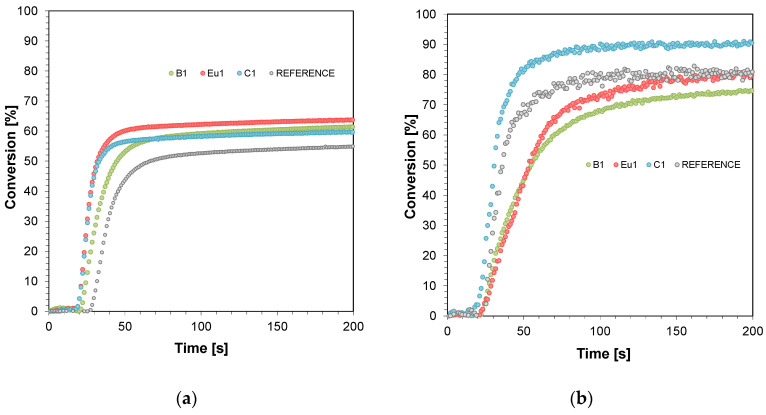
Polymerisation profiles based on TMPTA, IBOA, and BEDA monomers in a weight ratio of 1:2:7 (acrylate function conversion as a function of irradiation time) in the presence of different photoinitiating systems based on diphenyl(2,4,6-trimethylbenzoyl)phosphine oxide (TPO) and fluorescent dyes (0.1%) after irradiation with LED at 405 nm (0.4 mW/cm^2^): (**a**) in thin layers (25 μm) and (**b**) thick layers (0.5 mm). Irradiation starts at t = 10 s.

Real-time FT-IR was used to monitor the radical photopolymerisation, determining the final stages of monomer conversion in the resin. The radical photopolymerisation process was monitored by observing the disappearance of acrylate monomer bands with a wave number of approximately 1634 cm^−1^ (thin layer) and 6164 cm^−1^ (thick layer) corresponding to the disappearance of double bonds in the monomers tested (Figures S1–S8). A process-initiating LED was used as the light source, with an emission maximum of 405 nm (λ_max_ = 405 nm). Figure 1 presents polymerisation profiles based on TMPTA, IBOA, and BEDA monomers in a weight ratio of 1:2:7 (acrylate function conversion as a function of irradiation time) in the presence of different photoinitiating systems based on diphenyl(2,4,6-trimethylbenzoyl)phosphine oxide (TPO) and fluorescent dyes (0.1%) after irradiation with LED at 405 nm (0.4 mW cm^−2^) in thin layers (25 μm) and thick layers (0.5 mm).

Comparing the photopolymerisation results performed in thin layers shows significant differences in the achieved conversion rates (Figure 1a). In each case, the addition of the tested dye significantly increased the monomer conversion rate and the photopolymerisation rate (Table 1). Therefore, it can be seen that the tested dyes (BODIPY, Eu1, and Coumarin 1) can act as “photosensitizers”. However, for thick films, monomer conversion decreased slightly for the BODIPY dye and the europium complex (Figure 1b). In the case of photopolymerisation of thick films, overheating of the sample occurs during the radical photopolymerisation process, which can have an adverse effect on the speed of the photopolymerisation process [47]. This may also be related to the significant fluorescence intensity, which would limit light penetration deep into the formulation. On the other hand, Coumarin 1, which has an absorption band strongly covering the maximum emission of light source, behaved as the strongest photosensitizer of those presented.

### 2.2. Determination of Polymerisation Shrinkage Using an Anton Paar Apparatus

Polymerisation shrinkage is very common during radical photopolymerisation processes [27]. Therefore, the effect of the addition of dyes on the polymerisation shrinkage of the compositions tested was examined.

**Figure 2:**
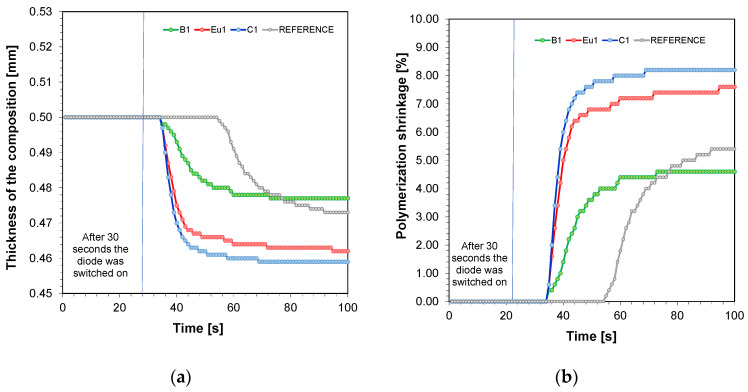
(**a**) Thickness of the compositions versus exposure time for a formulation containing TMPTA, IBOA, and BEDA monomers in a weight ratio of 1:2:7, TPO initiator at 1 wt%, and fluorescent dyes at 0.1 wt%; (**b**) polymerisation shrinkage versus exposure time for a formulation containing TMPTA, IBOA, and BEDA monomers in a weight ratio of 1:2:7, TPO initiator at 1 wt%, and fluorescent dyes at 0.1 wt%.

The photorheological test was used to determine the polymerisation shrinkage. The detailed process of the investigation is presented in the experimental section. Figure 2a shows the thickness of the compositions versus exposure time for a formulation containing TMPTA, IBOA, and BEDA monomers in a weight ratio of 1:2:7, TPO initiator at 1 wt%, and fluorescent dyes at 0.1 wt%. In contrast, Figure 2b shows the calculated polymerisation shrinkage according to Equation (2). 

When comparing the formulations in terms of thickness, the greatest change was observed for the formulation with Coumarin 1 added (Figure 2a). This corresponds to the greatest polymerisation shrinkage during the photopolymerisation process (Figure 2b) (Table 2). This is probably due to the fastest polymerisation reaction when this dye is applied to thick films, as confirmed by the FT-IR studies. On the other hand, both thickness and volume shrinkage were lowest for the BODIPY dye. This result is also analogous to the results obtained by FT-IR, where in thick films the photopolymerisation for BODIPY formulations had the lowest conversion and was slower than the others.

### 2.3. Three-Dimensional Printing Experiment

**Figure 3:**
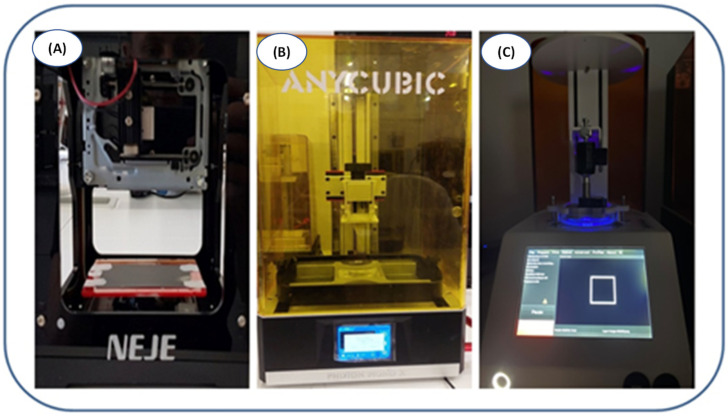
(**A**) NEJE laser engraver printer; (**B**) Photon Mono X printer; (**C**) LumenX printer.

A number of application tests were performed using various 3D printers during the study (Figure 3). Prints were made from resins prepared in a manner analogous to FT-IR and photorheological measurements. All prints were performed under aerobic conditions. All print designs were made using software dedicated to each type of printer. Formulations used during the 3D printing studies were analogous to those in the FT-IR measurements.

The laser engraver printer experiments used a 405 nm laser diode. A few drops of the composition were applied to the print field (microscope slide) and then given a laser exposure of 1000 mW at burning time = 1 ms.

For the Photon Mono X printer—commonly used in SLA printing technologies—resins were applied for bathtubs lined with non-stick foil. Printing was conducted using a 405 nm diode. The exposure time for the first two layers was 30 s and each subsequent layer was 12 s.

For the last printer, LumenX, which is dedicated to obtaining polymeric hydrogel materials, the printing was performed analogically to that of the Anycubic printer. In this case, the layer exposure time was 15 s and each subsequent layer exposure time was 3 s. Moreover, a 405 nm light source was used, and the printer power was set at 25% of the total power.

**Figure 4:**
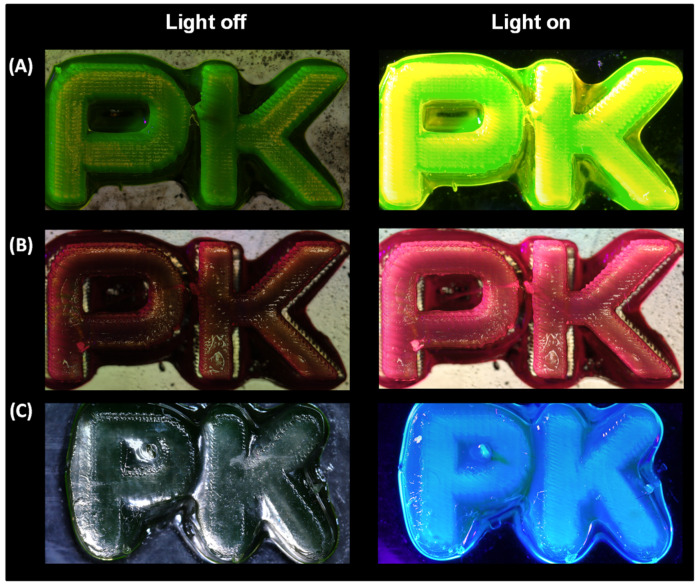
Print comparison obtained by NEJE laser engraver printer. Comparison was made with light off and light on for: (**A**) BODIPY; (**B**) Eu1; and (**C**) Coumarin 1.

A laser engraver printer was used to print the PK pattern (Figure 3). Each of the used dyes produced a final print characterised by the appropriate, desired fluorescence. When BODIPY and Eu1 were used, prints had good resolution, edges were clearly defined, and material shrinkage was low (Figure 4). For the Coumarin 1 printout, the resolution was significantly lower than for the others. No outlined shape was observed. The edges of the print were blurred. Additionally, the composition outside the assumed print area polymerised. Relating this result to the FT-IR tests, it can be observed that in the case of Coumarin 1, the unsatisfactory result may be due to the photopolymerisation process being too fast.

**Figure 5:**
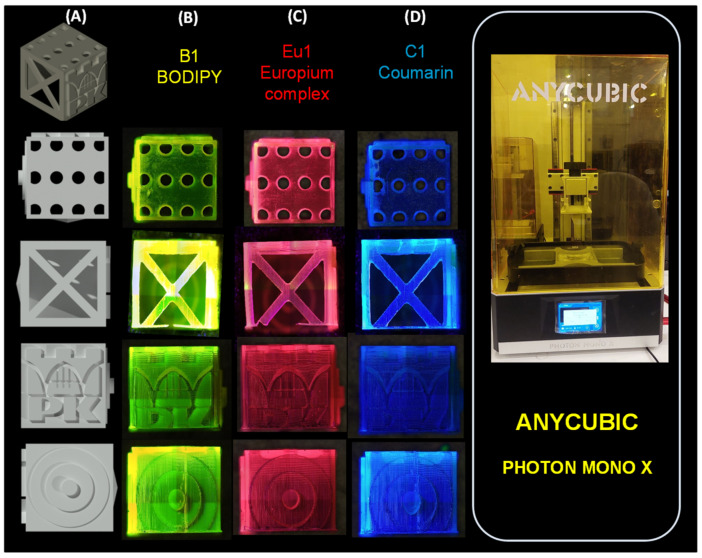
(**A**) Three-dimensional cube model made in AutoCAD 2020; print comparison obtained by Photon Mono X printer. Comparison was made with light on (366 nm) for: (**B**) B1; (**C**) Eu1; and (**D**) C1. Three-dimensional objects were observed using the OLYMPUS DSX1000 optical microscope.

For the printouts obtained with the Photon Mono X printer (Figure 5), the results obtained are similar to each other. Only in the case of Coumarin 1, a larger but still small polymerisation shrinkage can be observed. However, all printouts obtained with this printer were satisfactory.

**Figure 6:**
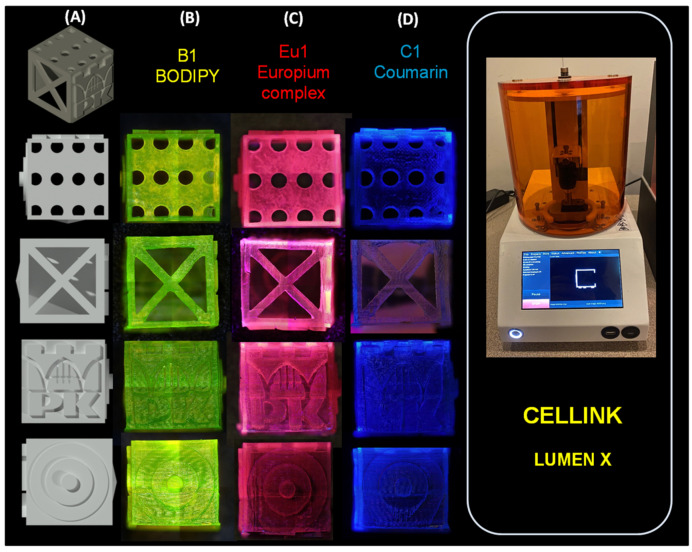
(**A**) Three-dimensional cube model made in AutoCAD 2020; print comparison obtained by LumenX printer. Comparison was made with light on (366 nm) for: (**B**) BODIPY; (**C**) Eu1; and (**D**) Coumarin 1. Three-dimensional objects were observed using the OLYMPUS DSX1000 optical microscope.

Analysing the prints with the last printer (LumenX), it can be seen that for dyes Eu1 and Coumarin 1, the polymerisation shrinkage was high; the obtained patterns were blurred. The highest resolution was achieved for BODIPY, which may be related to the fact that the photopolymerisation process with this dye was the slowest, so the shrinkage was small (Figure 6). These results are analogous to the FT-IR and photorheology studies performed previously. It is also worth mentioning that printouts created with this printer were characterised by different mechanical properties than in other cases. Their structure was flexible, which is characteristic for hydrogel structures, for which the printer is dedicated.

The formulations tested during 3D printing behaved as we predicted based on the results obtained using FT-IR and photorheology methods (e.g., the highest shrinkage during polymerisation was obtained for the composition with Coumarin 1).

Nevertheless, in each case, prints with specific characteristic colours were obtained when exposed with a UV light lamp with a maximum emission of 366 nm: for the composition containing the BODIPY chromophore, a yellow print was obtained; for the composition containing the europium complex, a red print was obtained; and for the composition containing Coumarin 1, a blue print was obtained. Interestingly, in sunlight (Figures S9 and S10), only the print containing the BODIPY chromophore was green, while the prints with the europium complex and Coumarin 1 were transparent.

**Figure 7:**
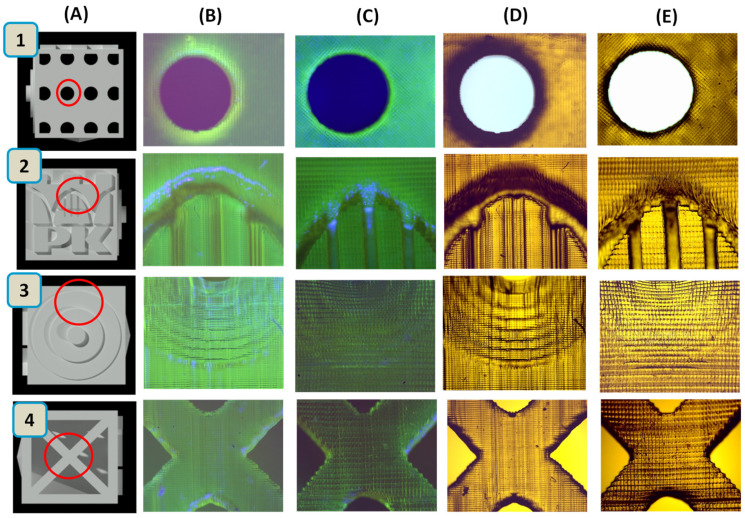
(**A**) Three-dimensional cube model made in AutoCAD; prints consist of BODIPY obtained by: (**B**) Anycubic Mono X printer in sunlight; (**C**) LumenX printer in sunlight; (**D**) Anycubic Mono X printer for irradiation with UV-LED at 366 nm; and (**E**) LumenX printer for irradiation with UV-LED at 366 nm. The 3D objects generated were observed using the Genetic Pro microscope, Delta Optical.

Figure 7 presents a comparison of prints containing the BODIPY chromophore from the LumenX and Anycubic Mono X printers both in sunlight and for irradiation with UV-LED at 366 nm. The 3D objects generated were observed using a Genetic Pro microscope from Delta Optical. It can be seen that prints from the Lumen X printer have a higher resolution. For the composition containing the fluorescent dyes europium complex and Coumarin 1, prints are included in the Supplementary Materials (Figures S11 and S12). Photos of real prints obtained by the Anycubic Mono X and LumenX printer in sunlight and under UV-LED at 366 nm are presented in Figures S13 and S14.

**Figure 8:**
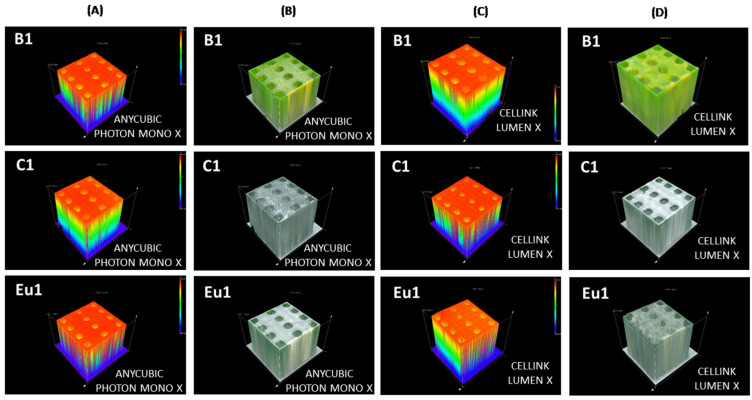
Three-dimensional spectra for cubes containing fluorophores B1, C1, Eu1 observed on the OLYMPUS DSX1000 optical microscope: (**A**) height of cubes made using the Anycubic Photon Mono X printer; (**B**) 3D view of cubes in sunlight made using the Anycubic Photon Mono X printer; (**C**) height of cubes made using the LumenX printer; (**D**) 3D view of cubes in sunlight made on the Anycubic LumenX printer.

It is worth highlighting that the cubes were 1 cm × 1 cm × 1 cm; their 3D spectra are presented in Figure 8.

## 3. Discussion

These studies confirmed that the addition of fluorescent dyes to the light-curing compositions containing a mixture of acrylate monomers improved the visual effects of the prints obtained. For the study, three dyes, [4-phenyl-2,6-bis(2-pyridyl)pyridine]tris [4,4,4-trifluoro-1-(4-nitrophenyl)butane-1, 3-dione]europium(III) (Eu1), 7-diethylamino-4-methylcoumarin (Coumarin 1), and B-1 4,4-difluoro1,3,5,7,8-pentamethyl-4-bora-3a,4a-diaza-s-indecene (BODIPY), were investigated. The addition of these dyes was dictated by the fact that these dyes exhibit strong fluorescence [48,49,50] and safety in application [51,52,53]. These dyes are widely used in biology, e.g., BODIPY is applied in biochemical labelling [54] and europium complex and coumarin are employed in live cell imaging applications [55,56].

Kinetic studies confirmed that the addition of fluorophores increased the rate of radical photopolymerisation and enhanced the final conversion rates. All the tested fluorophores accelerated the radical photopolymerisation process of thin films (in the order of 25 um), while photopolymerisation of thick films (0.5 mm) with the Coumarin 1 fluorophore improved the final conversion rates. That is, only Coumarin 1 exhibited an accelerating effect in this case. A slight slowing down of the photopolymerisation process was observed when the BODIPY chromophore and europium were applied. This may be due to the excessive fluorescence intensity coming from the investigated fluorophores. Therefore, the application of Coumarin 1 for 3D printing in light-curing compositions seems to be most preferable. Nevertheless, the studies showed that when Coumarin 1 was used, the highest polymerisation shrinkage was obtained during the photopolymerisation process. Polymerisation shrinkage is extremely undesirable in photopolymerisation processes, as it can cause a number of unwanted effects in 3D printing. Volumetric shrinkage, in particular, can induce strong internal stresses that can cause deformation of the material, which may even result in model breakage. Otherwise, volumetric shrinkage causes a decrease in the precision of the print model [40]. Although the final conversion rates using fluorophores such as BODIPY and europium complex were lower than when Coumarin 1 was used, prints with good resolution and lower polymerisation shrinkage were obtained.

Thus, a balance needs to be found between printing speed, the final conversion rates of the photopolymerisation process, and minimal volume shrinkage. Further research directions encourage the development of this topic with the application of other monomers, in particular cationic polymerising monomers which are characterised by significantly reduced polymerisation shrinkage. Indeed, the addition of Coumarin 1 to cationic polymerisation resins can lead to fluorescent prints with high resolution and low polymerisation shrinkage.

## 4. Materials and Methods

**Figure 9:**
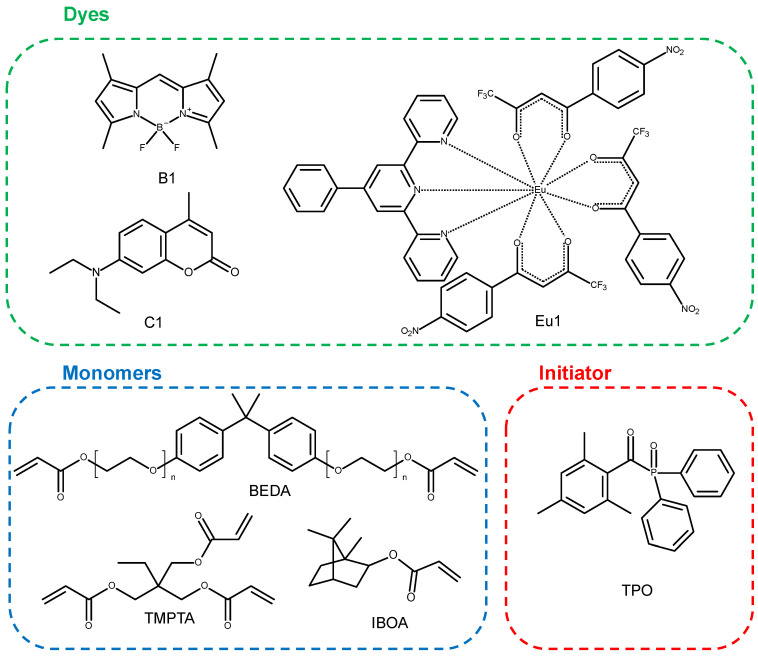
Structures of molecular fluorescent dyes, monomers, and initiator used for photopolymerisation processes.

The fluorescent dyes [4-phenyl-2,6-bis(2-pyridyl)pyridine]tris [4,4,4-trifluoro-1-(4-nitrophenyl)butane-1,3-dione]europium(III) (Eu1), 7-diethylamino-4-methylcoumarin (Coumarin 1, Alfa Aesar), and 4,4-difluoro1,3,5,7,8-pentamethyl-4-bora-3a,4a-diaza-s-indecene (BODIPY, B1) were used. The synthesis of the compound 4,4-difluoro1,3,5,7,8-pentamethyl-4-bora-3a,4a-diaza-s-indecene (BODIPY, B1) was described in a previous article [41]. Monomers used for the compositions were isobornyl acrylate (IBOA, IGM RESINS), bisphenol A ethoxylate diacrylate (BEDA, Sigma Aldrich, St. Louis, MO, USA), and trimethylolpropane triacrylate (TMPTA, Sigma Aldrich). The initiator diphenyl(2,4,6-trimethylbenzoyl)phosphine oxide (TPO, Sigma Aldrich) was used. The structures of the molecular fluorescent dyes, monomers, and initiator are presented in Figure 9.

### 4.1. Preparation of Samples for Monitoring the Photopolymerisation Processes by FT-IR

Thick film compositions were prepared for real-time FT-IR photopolymerisation studies. The compositions consisted of a suitable monomer trimethylolpropane triacrylate (TMPTA), bisphenol A ethoxylate diacrylate (BEDA), isobornyl acrylate (IBOA), and fluorescent dyes—BODIPY, Coumarin 1, and Eu1 accounting for about 0.1 wt% of the whole composition and diphenyl(2,4,6-trimethylbenzoyl)phosphine oxide (TPO) for 1.0 wt% in the monomer (Figure 9).

### 4.2. Monitoring the Photopolymerisation Processes by Real-Time FT-IR

The kinetics of radical photopolymerisation was studied by real-time FT-IR using a NICOLETTM FT-IR i10 spectrometer with a horizontal adapter (Thermo Scientific, Waltham, MA, USA).

Real-time FT-IR measurement consisted of placing a few drops of a composition based on TMPTA, BEDA, and IBOA monomers in a 0.5 mm thick ring using a plastic pipette. In addition, measurements were made in thin layers (25 μm). A drop of the composition was placed between two sections of the laminate. Attempts were made to maintain the same thickness of the sample. For thick layers, the special ring (0.5 mm) was used. The formulation was added dropwise to achieve a smooth surface. Compositions prepared in thick and thin layers were placed in a holder made of metal and then in a horizontal attachment attached to the spectrometer. Photopolymerisation measurements were carried out using a photopolymerisation time of 200 s. The experiments were conducted in a dark room, where only light emitting red radiation with a wavelength of λ_max_ = 405 nm was used as a light source. The real-time light source for the FT-IR method was a 405 nm (0.4 mW/cm^2^) M405L4 diode (Thorlabs Inc., Newton, NJ, USA), powered by a regulated DC2200 power supply (from Thorlabs Inc., Newton, NJ, USA). The UV-LED was activated 10 s after the start of spectral recording. The distance between the radiation source and the preparations was 2.1 cm.

Because the decrease of absorption of the peak area is directly proportional to the number of polymerised groups, the degree of conversion of the function group was calculated by measuring the peak area at each time of the reaction by using Equation (1):

(1) $$C_{FT-IR}\left[\%\right]=\left(1-\frac{A_{\mathrm{After}\;}}{A_{\mathrm{Before}\;}}\right)\cdot 100\%$$

where *A_Before_* is an area of the absorbance peak characteristic for the used monomer and type of photopolymerisation before the polymerisation process and *A_After_* is an area of the same absorbance peak, but after the polymerisation process.

The values of the characteristic absorbance peak for the studied monomers in the photopolymerisation process are given below. The acrylate group content (monomers TMPTA, BEDA, and IBOA) was tracked continuously in thick film conditions (0.5 mm) at about 6164 cm^−1^ and in thin films at 1634 cm^−1^ (Figures S1–S10).

### 4.3. Photorheological Assessment of Photo-Cured Compositions

Photorheological tests were performed using an Anton Paar rheometer (Physica MCR 302) equipped with a UV light curing system with a parallel plate geometry of 20 mm. The 405 nm emitting wavelength diode from Bluepoint LED eco (Honle, Germany) was used as the light source with an intensity of 0.4 mW/cm^2^. The intensity of the light was measured using a PM160 - Si Sensor power meter (from Thorlabs Inc., Tampa, FL, USA). In the experiment, the separation distance between the two plates was set at 0.2 mm, with a constant frequency of 10 Hz and a deformation amplitude of 1%. The light was switched on 30 s after the start of the measurement to stabilise the system.

The polymerisation shrinkage was calculated from Equation (2):

(2) $$Shrinkage\left[\%\right]=\left(1-\frac{T}{T_{Before}}\right)\cdot 100\%$$

where *T_Before_* is the thickness of the composition before exposure and *T* is the thickness of the composition during exposure

### 4.4. Three-Dimensional Printing Experiment

Laser recording and 3D printing experiments used a 405 nm, 100 mW/cm^2^ laser diode (spot size ~50 μm) for spatially controlled illumination (NEJE DK-8-KZ 1000 mW Laser Engraver Printer). Light-sensitive slides (2 mm thick) were mounted on a microscope slide and polymerised under air. Furthermore, prints were made using the LumenX (Cellink3D) and Photon Mono X (Anycubic) printers. Both printers were equipped with a diode emitting a wavelength of 405 nm.

### 4.5. Characterisation of the 3D Patterns by Fluorescent Microscopy

The 3D objects generated were observed using the OLYMPUS DSX1000 optical microscope and a Genetic Pro microscope, Delta Optical.

## 5. Conclusions

The investigated dyes showed good absorbing properties and exhibited fluorescent properties. Therefore, it is possible to use them as additives in 3D printing to produce prints that also exhibit fluorescent properties. Using the prepared compositions, prints were obtained in an efficient manner. All obtained printed patterns exhibited good spatial resolution. From the systems tested, based on FT-IR and photorheology studies, it can be concluded that the system containing Coumarin 1 polymerised most rapidly and efficiently. However, when relating this to the print studies carried out, it can be seen that the prints with Coumarin 1 are the worst in terms of physical properties. This is probably due to the fact that when the polymerisation process is too fast, too much shrinkage of the material occurs, which is associated with a deterioration of the print quality. In order to prevent the phenomenon described above, it would be necessary to choose appropriate printing conditions (slower) for this particular composition. In summary, however, the performed studies indicate that fluorescent prints can be obtained from the proposed systems.

## 6. Patents

The synthesis of the BODIPY chromophore is described in the National Patent Pat.238234, granted 13 May 2021, previously patent application P.431489 with priority date 16 October 2019 (authors: Monika Topa, Joanna Ortyl, Mariusz Galek; title: “New iodonium salts, production method and applications”).

## Figures and Tables

**Table 1:** Summary of acrylate functional group conversions for radical photopolymerisation processes, following LED at 405 nm irradiation (0.5 mW/cm^2^) using different dyes.

Dyes	Thin Layers (25 μm)			Thick Layers (0.5 mm)		
	Conversion (%)	dC_FT-IR_/dt (s^−1^)	t_idn_ (s)	Conversion (%)	dC_FT-IR_/dt (s^−1^)	t_idn_ (s)
Reference	54.9	1.47	18.8	81.8	2.19	15.3
BODIPY	61.4	1.75	14.2	74.5	1.30	15.2
Eu1	63.6	2.50	12.3	81.0	1.29	20.0
C1	59.7	2.55	13.8	89.4	3.06	15.8

dC_FT-IR_/dt—photopolymerisation rate; t_ind_—induction time.

**Table 2:** Comparison of composition thickness and polymerisation shrinkage for the studied formulations.

Dyes	Thickness of the Composition (mm)	Polymerisation Shrinkage (%)
Reference	0.473	5.4
BODIPY	0.477	4.6
Eu1	0.462	7.6
C1	0.459	8.2

## Data Availability

Not applicable.

## Supplementary Materials

The following supporting information can be downloaded at: https://www.mdpi.com/article/10.3390/ijms231810470/s1.
