# Supplementary material for: Novel Formulations Containing Fluorescent Sensors to Improve the Resolution of 3D Prints
Source: Int J Mol Sci. 2022 Sep 9;23(18):10470. doi: 10.3390/ijms231810470 (PMC9504832; doi:10.3390/ijms231810470)
Supplement: Supplementary file 1 [file ijms-23-10470-s001.zip › ijms-1876338-supplementary.pdf]

International Journal of
Molecular Sciences

MDPI

Suplementary Materials

Novel Formulations Containing Fluorescent Sensors to Improve the Resolution of 3D Prints

Monika Topa-Skwarczyńska [1,2,*], Andrzej Świeży [1,3], Dominika Krok [1], Katarzyna Starzak [1], Paweł Niezgoda [1,2], Bartosz Oksiuta [1], Weronika Wałczyk [1] and Joanna Ortyl [1,2,3,*]

[1] Department of Biotechnology and Physical Chemistry, Faculty of Chemical Engineering and Technology, Cracow University of Technology, Warszawska 24, 30-155 Kraków, Poland
[2] Photo4Chem Ltd., Lea 114, 30-133 Kraków, Poland
[3] Photo HiTech Ltd., Bobrzyńskiego 14, 30-348 Kraków, Poland
* Correspondence: monika.topa@doktorant.pk.edu.pl (M.T.-S.); jortyl@pk.edu.pl (J.O.)

Abstract: The rapid development of Industry 4.0 and its growing impact on the global economy is prompting researchers to explore technologies that can find real applications in such processes. 3D printing in SLA and DLP technologies, which has recently experienced a period of extremely rapid development, perfectly fits into the assumptions outlined by the solutions of Industry 4.0. Stereolithography technologies, which use light radiation of 405 nm wavelength to build an element, bring many applications. Due to high resolution, speed of modeling and printing process, these elements are ideal for prototyping technology, which is currently the basis for most industries. In addition, the possibility of modifying the parameters of models in terms of physicochemical properties opens a number of other solutions in which printed materials can find their application, such as medicine or dentistry. In the following paper, the effects of dyes such as BODIPY, europium complex and Coumarin 1 added to light-cured compositions polymerising according to the radical mechanism on the photopolymerisation process speed, polymerisation shrinkage and the final properties of the printouts were investigated. The kinetics of photopolymerisation of light-cured materials using Real-Time FT-IR methods, as well as printouts that tangibly demonstrate the potential application of 3D printing technology in Industry 4.0 were examined. Polymerisation shrinkage was also measured using an Anton Paar apparatus. These studies showed that the addition of dyes has an effect on obtaining fluorescent prints with good resolution.

Keywords: photopolymerisation; reology; Real-Time FT-IR; advanced materials; 3D printing

1. Before and after Polymerisation Spectra for the Photopolymerisation of Thin Films (25 μm) of a Formulation Containing Fluorescent Dyes

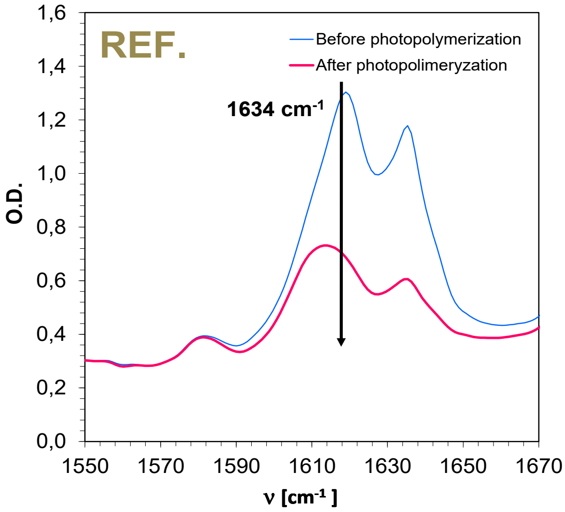

Figure S1. FT-IR spectra before and after photopolymerization of TMPTA, IBOA, BEDA monomers in a weight ratio of 1:2:7 under Vis-LED 405 nm irradiation (20 mW/cm^2) in composition with TPO (1% wag.) as the photoinitiator in thin layers (25 μm).

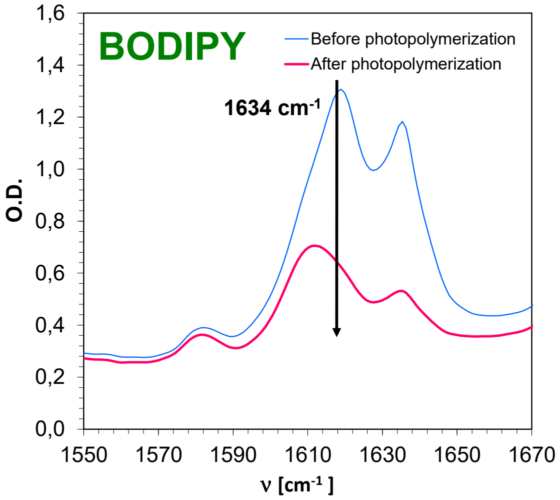

Figure S2. FT-IR spectra before and after photopolymerization of TMPTA, IBOA, BEDA monomers in a weight ratio of 1:2:7 under Vis-LED 405 nm irradiation (20 mW/cm^2) in composition with TPO (1% wag.) as the photoinitiator and fluorescent dyes BODIPY (0.1%) in thin layers (25 μm).

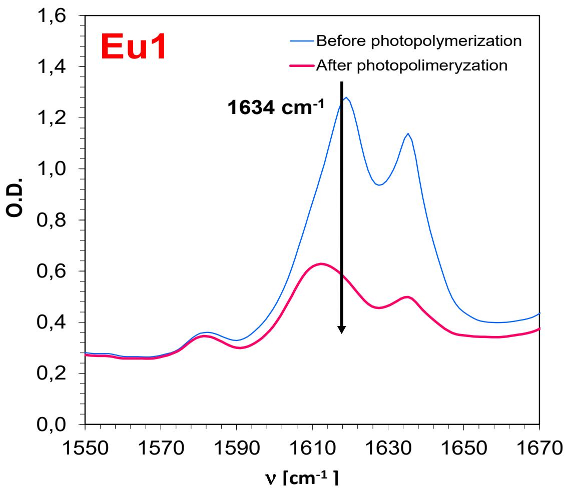

Figure S3. FT-IR spectra before and after photopolymerization of TMPTA, IBOA, BEDA monomers in a weight ratio of 1:2:7 under Vis-LED 405 nm irradiation (20 mW/cm^2) in composition with TPO (1% wag.) as the photoinitiator and fluorescent dyes Eu1 (0.1%) in thin layers (25µm).

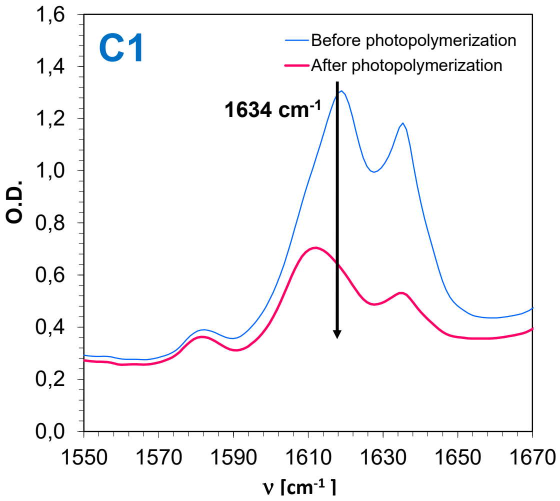

Figure S4. FT-IR spectra before and after photopolymerization of TMPTA, IBOA, BEDA monomers in a weight ratio of 1:2:7 under Vis-LED 405 nm irradiation (20 mW/cm^2) in composition with TPO (1% wag.) as the photoinitiator and fluorescent dyes Coumarin 1 (0.1%) in thin layers (25µm).

2. Before and after Polymerisation Spectra for the Photopolymerisation of Thick Films (0.5 mm) of a Formulation Containing Fluorescent Dyes

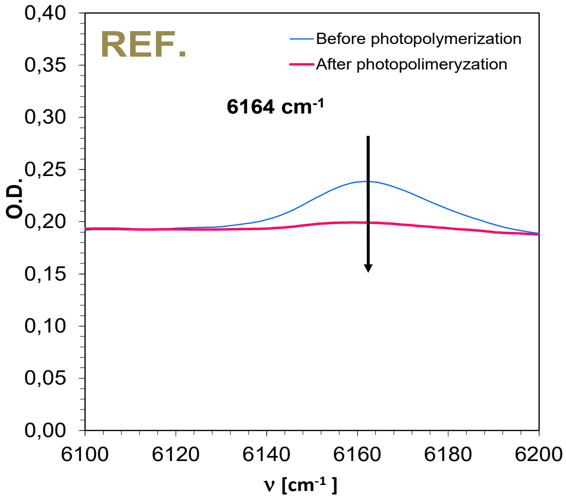

Figure S5. FT-IR spectra before and after photopolymerization of TMPTA, IBOA, BEDA monomers in a weight ratio of 1:2:7 under Vis-LED 405 nm irradiation (20 mW/cm^2) in composition with TPO (1% wag.) as the photoinitiator in thick layers (25 µm).

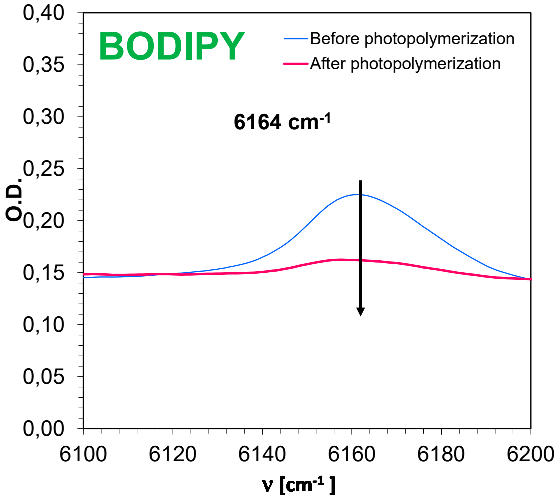

Figure S6. FT-IR spectra before and after photopolymerization of TMPTA, IBOA, BEDA monomers in a weight ratio of 1:2:7 under Vis-LED 405 nm irradiation (20 mW/cm^2) in composition with TPO (1% wag.) as the photoinitiator and fluorescent dyes BODIPY (0.1%) in thick layers (25 µm).

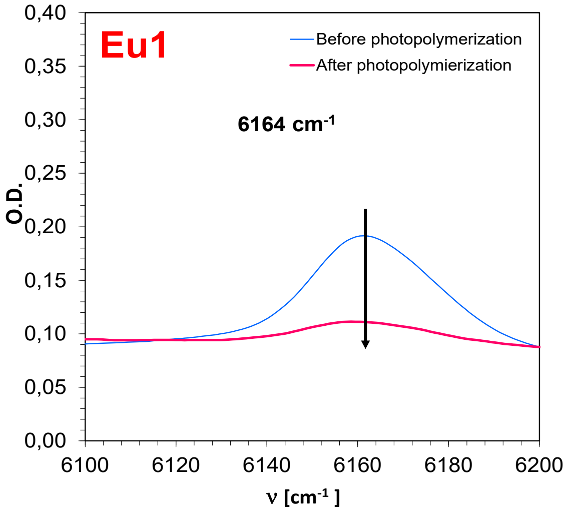

Figure S7. FT-IR spectra before and after photopolymerization of TMPTA, IBOA, BEDA monomers in a weight ratio of 1:2:7 under Vis-LED 405 nm irradiation (20 mW/cm^2) in composition with TPO (1% wag.) as the photoinitiator and fluorescent dyes Eu1 (0.1%) in thick layers (25 μm).

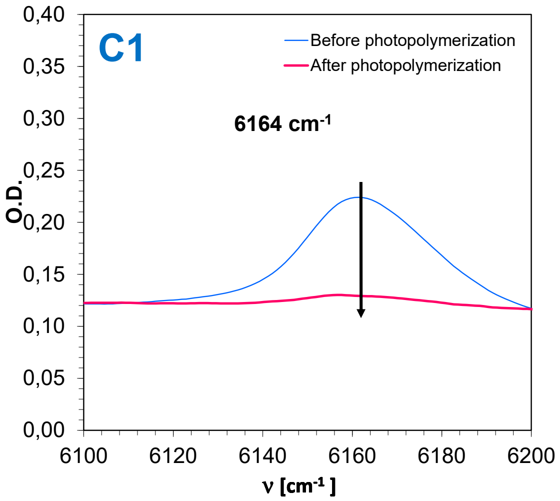

Figure S8. FT-IR spectra before and after photopolymerization of TMPTA, IBOA, BEDA monomers in a weight ratio of 1:2:7 under Vis-LED 405 nm irradiation (20 mW/cm^2) in composition with TPO (1% wag.) as the photoinitiator and fluorescent dyes Coumarin 1 (0.1%) in thick layers (25 μm).

3. 3D Printing Experimental

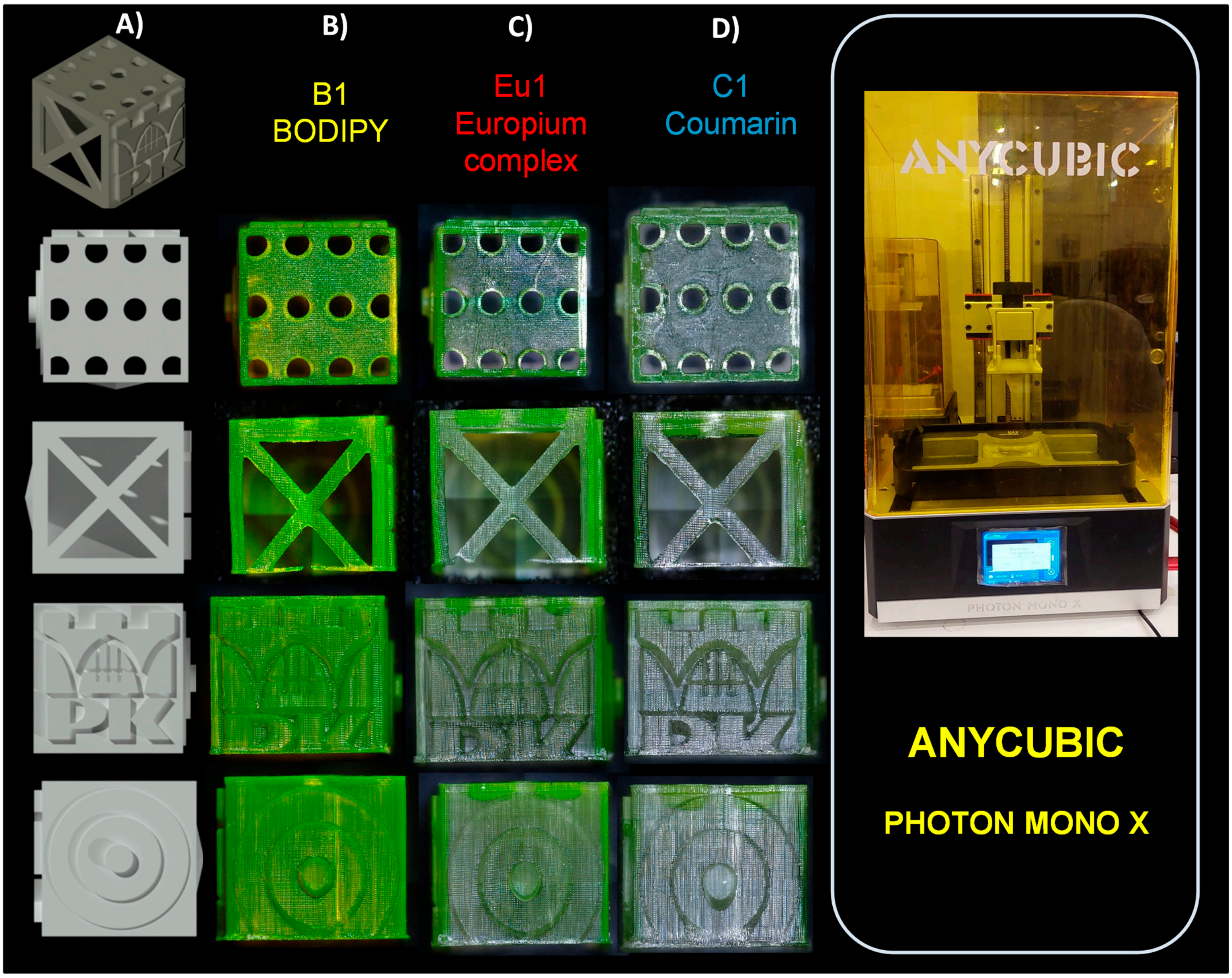

Figure S9. (**A**) 3D cube model made in Autocad; Print comparison obtained by Photon mono X printer. Comparison was made with light off (sunlight) for: (**B**) BODIPY; (**C**) Eu1; (**D**) Coumarin 1.

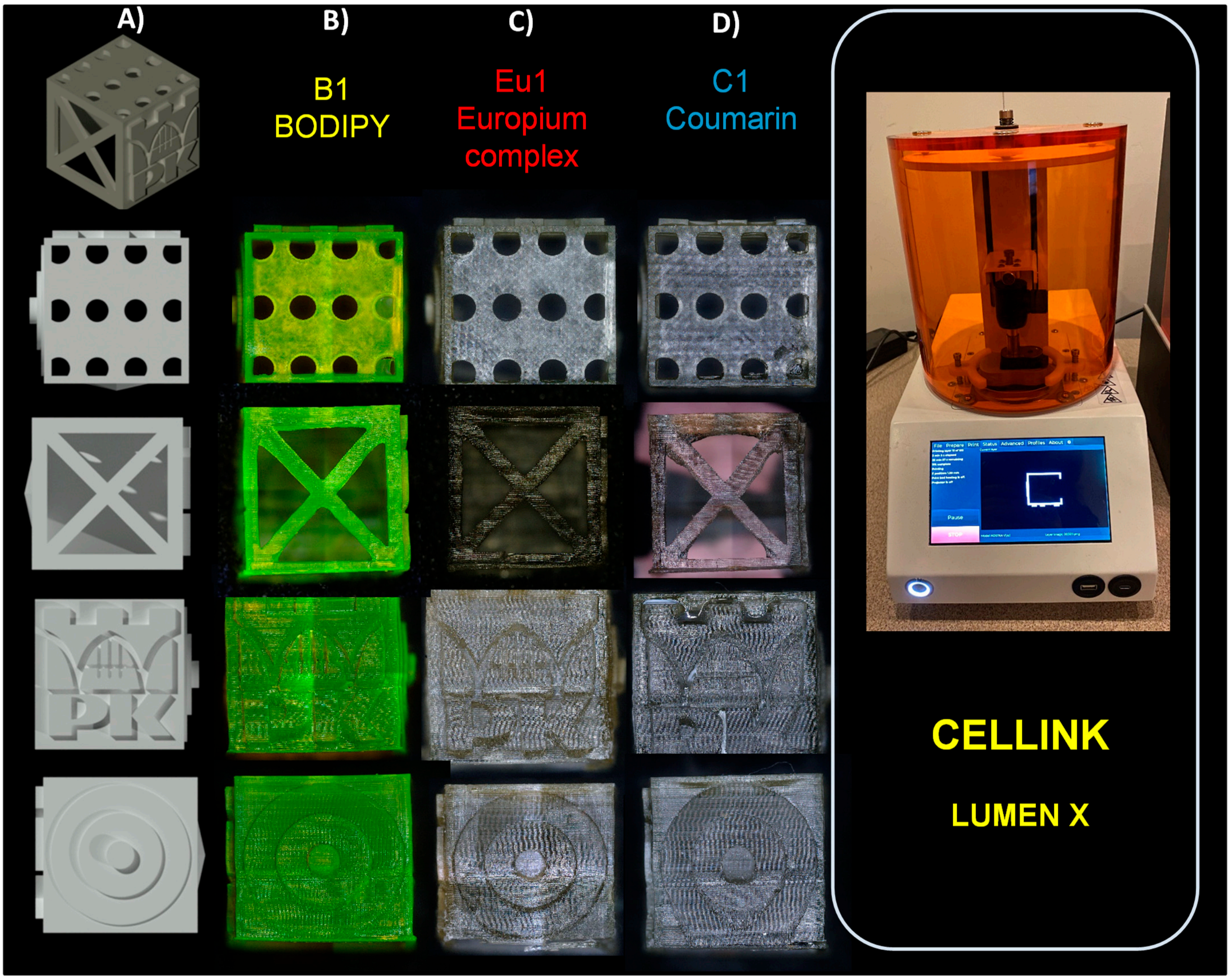

Figure S10. (**A**) 3D cube model made in Autocad; Print comparison obtained by LumenX printer. Comparison was made with light on (366 nm) for: (**B**) BODIPY; (**C**) Eu1; (**D**) Coumarin 1.

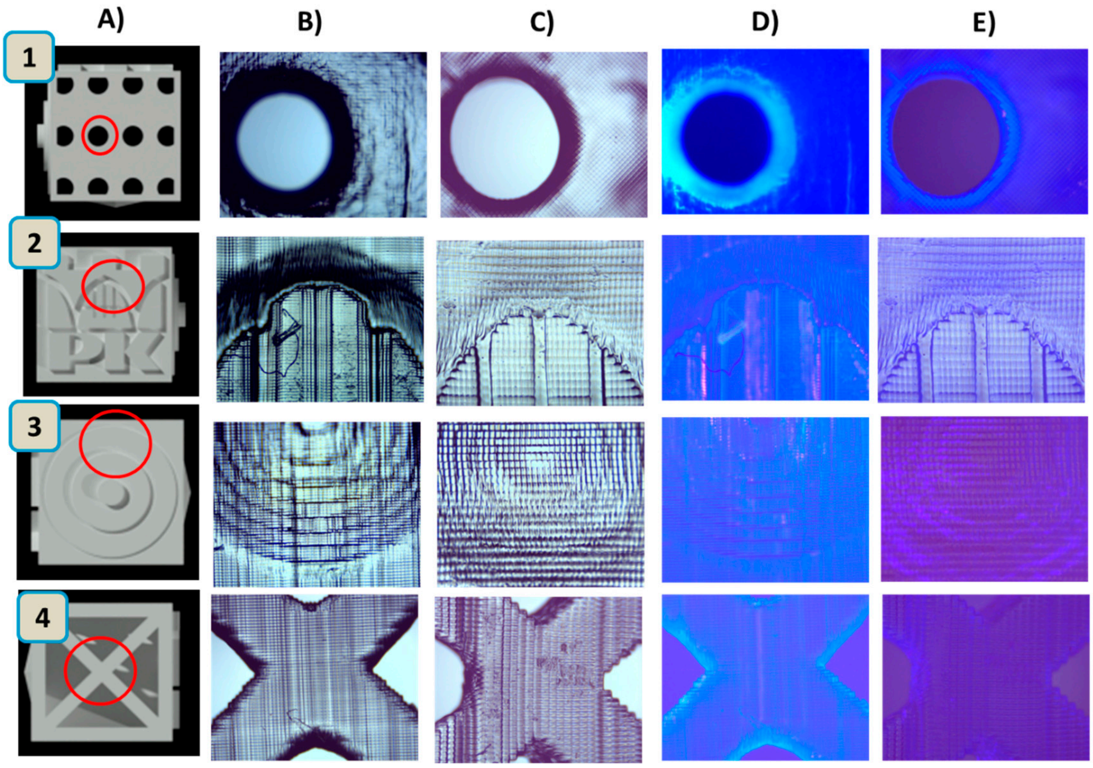

Figure S11. (**A**) 3D cube model made in Autocad; Print consist of Coumarin 1 obtained: (**B**) by Anycubic Mono X printer in sunlight; (**C**) by LumenX printer in sunlight; (**D**) by Anycubic Mono X printer for irradiation with UV-LED 366nm; (**E**) by LumenX printer for irradiation with UV-LED 366nm. The 3D objects generated were observed using the Genetic Pro microscope, Delta Optical.

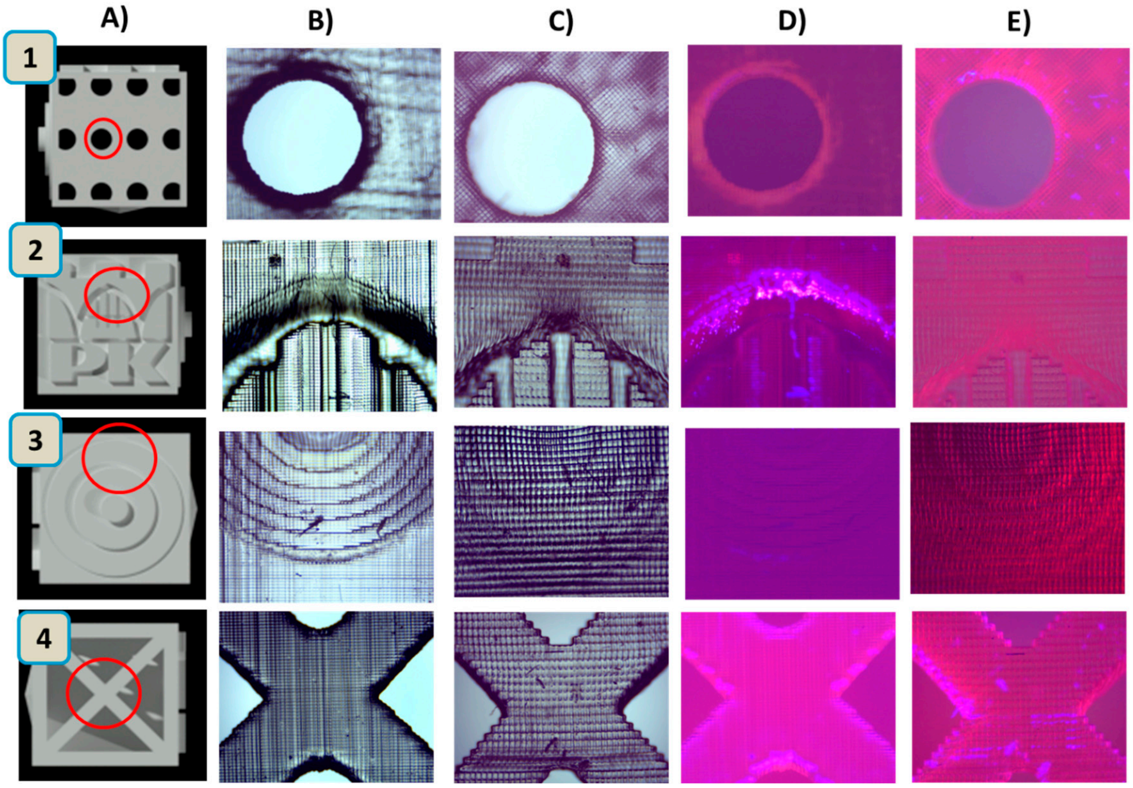

Figure S12. (**A**) 3D cube model made in Autocad; Print consist of Europium complex obtained: (**B**) by Anycubic Mono X printer in sunlight; (**C**) by LumenX printer in sunlight; (**D**) by Anycubic Mono

X printer for irradiation with UV-LED 366nm; (**E**) by LumenX printer for irradiation with UV-LED 366nm. The 3D objects generated were observed using the Genetic Pro microscope, Delta Optical.

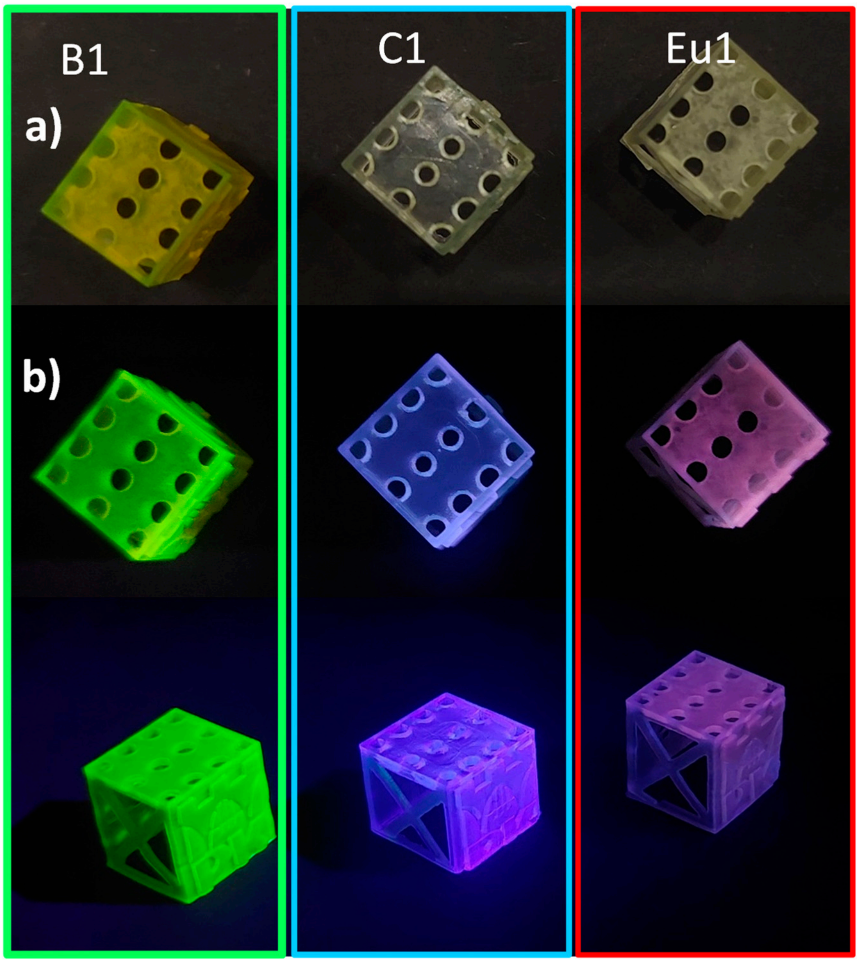

Figure S13. Prints obtained by Anycubic Mono X printer: (**a**) in sunlight; (**b**) with UV-LED 366nm.

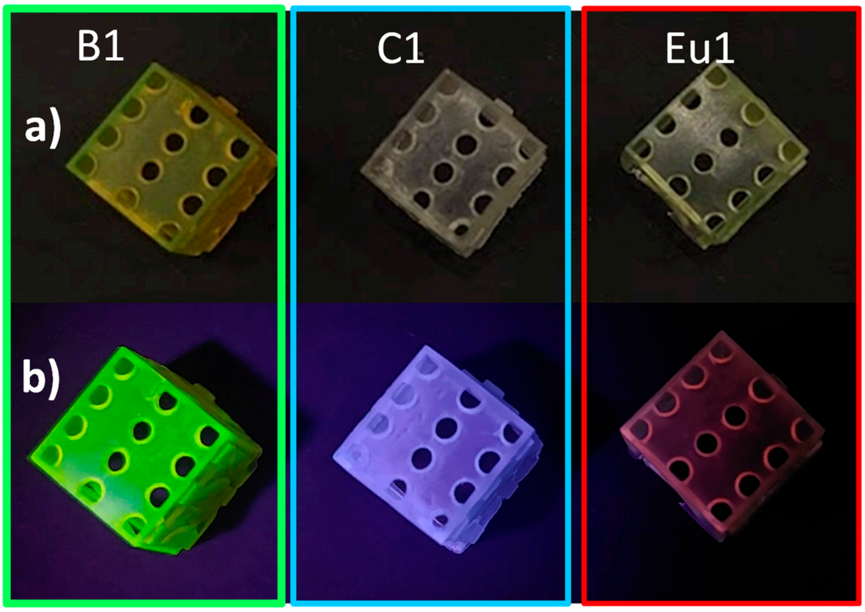

Figure S14. Prints obtained by LumenX printer: (**a**) in sunlight; (**b**) with UV-LED 366nm.
